# Multiple climate-related stressors in the tropics and beneficial changes in northern latitudes will mostly have emerged before 2050

**DOI:** 10.1371/journal.pone.0293551

**Published:** 2025-06-17

**Authors:** Audrey Brouillet, Benjamin Sultan

**Affiliations:** ESPACE-DEV, Univ Montpellier, IRD, Univ Guyane, Univ Reunion, Univ Antilles, Univ Avignon, Maison de la Télédétection, Montpellier, Cedex, France; Arab Academy for Science Technology and Maritime Transport, EGYPT

## Abstract

Consequently to global warming, multi-sectoral impacts are observed and should intensify in the future, affecting sectors of water resources, agriculture, weather extremes and health. Related projected change signs, their possible emergences from the historical variability, and how these emergences may cumulate in time and space could thus result in severe risks or benefits for local populations. Using the world’s largest cross-sectoral climate-related impact multi-model simulations database Inter-Sectoral Impacts Model Intercomparison Project (ISIMIP), here we quantify for the first time the Time of Emergence (TOE) of historical and future simulated changes in multiple climate-related indicators at a global scale. We assess how both adverse changes (i.e. stressors) and beneficial changes (i.e. benefits) could cumulate during the century. Based on ISIMIP2b (ISIMIP phase 2b) and a low mitigation future scenario (RCP6.0), we find that many land areas are characterized by a multi-model TOE earlier than 2020 for the majority of the 10 analyzed multi-sectoral indicators. This illustrates an already reached new equilibrium-state resulting from global warming for many sectors including hydrology, agriculture and weather extremes. However, the TOE varies depending on the region and the sectoral indicator, encompassing both projected stressors and beneficial changes. The largest number of cumulated emergences of cross-sectoral stressors is projected in the tropics and include detected TOE for higher heat stress extremes, and declining crop yields. Conversely, northern mid- and high- latitudes experience the greatest number of cumulated emergences of beneficial changes, primarly associated with future crop yields increase. These cumulative emergences of stressors (in the tropics) and beneficial changes (in northern latitudes) reach their peak before 2050, indicating an early impact and related emergence on multiple sectors. Nevertheless, substantial uncertainties are exhibited in this multi-model assessment of TOE, particularly in the tropics due to a *cascade of uncertainties* combining climate models, impacts models, aggregated cross-sectoral impacts, and TOE detection. This study brings time constrains of multiple climate-related changes. It particularly highlights the heightened and early cumulated stressors projected in tropical regions that may further exacerbate disparities and inequalities with northern latitudes. These results also confirm the tropics paradox where fewer greenhouse gas emissions correspond to more adverse impacts from global warming. Without further mitigation and adaptation strategies, vulnerable socio-economic conditions and limited resources in these areas will amplify the negative consequences arising from early and cumulative cross-sectoral emergences, both for populations and ecosystems.

## Introduction

Carbon emissions from burning fossil fuel is one of the causes of the observed increase in atmospheric temperature and resulting climate change [1]. As global mean temperature is projected to continue rising during the 21st century, resulting impacts are concurrently projected to intensify in many major sectors such as agriculture [2–5], hydrology and water resources [6–8], weather hazards/extremes [9, 10] and health [11–14]. In the associated research field, two major climate issues are explored to assess possible ecosystems and population exposures to these resulting risks: (1) how climate modifications may emerge from a former climate state, and (2) how diverse climate-related impacts can cumulate and/or co-occur in space and time.

Emergence of climate modifications is mostly analysed through the Time of Emergence (TOE) quantification, i.e. the moment when a new climate regime is statistically different enough from a given reference. TOE has been mostly assessed for temperature metrics, more sporadically for precipitation and recently for related impacted sectors. Observed changes in hot extreme temperatures are shown to have already emerged in many regions, with slightly later emergences than changes in seasonal means [15]. Hot extremes have also emerged earlier within the tropics than in mid- and high- latitudes due to an inherently lower variability in low latitudes [16]. In climate simulations from the 5th version of the Coupled-Models Intercomparison Project (CMIP5 [17]), a mean multi-models TOE of 2020 for summer cold records and a mean TOE of 2030 for the summer warm records are found under the large emissive RCP8.5 scenario, with the quasi-disappearance of cold records by 2100 [18]. Concerning rainfall, wet extremes are mainly projected to emerge in mid- and high- northern latitudes during the coming decades in CMIP5 models under the RCP8.5 scenario, despite large natural inter-annual and multi-model variabilities [15]. TOE analysis of seasonal precipitation changes under RCP8.5 indicate that emergences will occur before 2040 for significant changes and “hotspots” in Africa including the projected October-to-March wettening in East Africa or the April-to-September drying in South Africa [19]. Regional analyses have emphasized a projected reduced occurrence of wet days in West Sahel, resulting in a simulated dryer climate emergence in 2028–2052 in CMIP5 models [20]. In East Sahel, a wetter precipitation regime (more very wet days occurrences) is likely to emerge before 2054. TOE is considered as a critical measure for risk assessment in many sectors [16, 18, 20, 22]. In particular, the study of [Jägermeyr *et al*. 2021] [23] has focused on the emergence of agriculture modifications under warming projections. It shows largest significant adverse yield decreases for maize, soybean and rice in the latest CMIP6 than previously simulated in CMIP5,but more wheat yield due to higher atmospheric CO2 concentration. These impacts are projected to emerge before 2040 in CMIP6 for several main producing regions. These results suggest that major breadbasket regions could face distinct anthropogenic climatic risks earlier than previously anticipated [23].

Along with these TOE approaches, previous studies have focused on possible cumulative cross-sectoral impacts of global warming. According to various methodologies and observations, it was shown that global warming has already more than doubled both the global land area and the global population annually exposed to multiple extreme events [24]. In the future, global population exposure to severe impacts and risks including changes in drought intensity, water stress index, cooling demand or crop yields is projected to double or triple between +1.5°C and +3°C, with 85% –95% of this global exposure located in Asian and African regions [24–26]. Multiple impacts of climate hazards such as mean warming, heatwaves, drought, floods, wildfires or changes in natural land cover are also traceable on human-related sectors such as human health, water, food, economy, infrastructure or security [27]. Nevertheless, many papers have exhibited large uncertainties related to these cross-sectoral climate impacts and population exposure analysis. It was shown that larger uncertainties arise from the impact models than from the climate models [28], and the spread across different impact models seems to be a major component of climate impacts simulations uncertainty [29].

UNEP reports [30, 31] indicate that global warming adaptation actions locally and/or nationally focus on related water, agriculture, ecosystems and cross-cutting sectors, and thus primarily address flooding, drought, and rainfall variability. However, although adaptation implementation is increasing to face global warming, it does not keep up with increasing climate-related impacts intensity, frequency and timings. Planned actions could be even outstripped by accelerating climate changes and related impacts, which would further widen the adaptation implementation gap [31]. To support prioritisation of mitigation and adaptation solutions, it is thus of a major importance to address questions on the direction and scale of cross-sectoral changes, and on the timing and location of the worst climate extremes and related impacts [32, 33].

In this context, this paper proposes to combine for the first time both cumulative cross-sectoral climate-related stressors and emergences assessments, in order to quantify the corresponding time of emergence (TOE) of such cross-sectoral indicators. More specifically, we seek to identify the spatial distributions and the timings of these climate-related emergences affecting many major sectors, and determine how these both resulting impacts and TOE could cumulate and co-occur, which would result in a new concept of climate risks. We select a set of 10 climate-related indicators to illustrate major sectors that significantly affect populations (i.e. agriculture, hydrology, weather extremes, fires). This challenge is addressed by analysing the largest cross-sectoral synthesis of diverse multi-sectoral impacts of global warming, i.e. the Inter Sectoral Impact Model Intercomparison Project phase 2b (ISIMIP2b) [29], and we analyse these impact data simulations under the historical and the low mitigation greenhouse gas future scenario, i.e. the Representative Concentration Pathways 6.0 (RCP6.0) [34]. RCP6.0 indeed provides the largest amount of impact simulations in ISIMIP2b, while allowing stronger response of climate-related impacts compared to higher mitigation future scenarios. It also allows an assessment of cross-sectoral multi-impacts of high levels of warming [29] under a plausible future in terms of needed fossil fuel to fit along with corresponding high warming greenhouse gas emissions [35, 36].

The present article describes our correspondind findings using the following structure: i) a material and methods section describing the datasets analysed, the Time of Emergence (TOE) calculations and a description of each assessed sectoral indicator, ii) a section presenting the results, including the TOE spatial patterns per impact indicator, cumulated cross-sectoral emergences, and a regional assessment of multi-model uncertainties, iii) a discussion to put our findings into a broader context and iv) the conclusion.

## Materials and methods

### Multi-sectoral ISIMIP data

The Inter Sectoral Impact Model Intercomparison Project (ISIMIP) is a cross-sectoral synthesis of the differential impacts of climate change, including the associated uncertainties [29]. Different simulation protocols are defined as a set of common simulation scenarios based on the focus topic (3-4 protocols). In the present study, we analyse projections from the ISIMIP phase 2b since it was the most recent completed multi-model and multi-sectoral database at the time of writting this manuscript (ISIMIP3 currently in progress). The ISIMIP2b protocol considers impacts on different sectors at the global and regional scales, and provides a large database of process-based climate-impact-model output produced in a multisectoral/multimodel framework.

Simulations analyzed from ISIMIP2b are based on the same climate and socioeconomic input data, but Global Impact Models (GIMs [25]) differ from a sector to another [29]. In this work, we analyse all available ISIMIP2b simulation per sector, i.e. all possible GIM x Earth System Model (ESM) combinations per indicator. This allows the quantification of multi-model uncertainties and thus provide a robust multi-model representation of each indicator future changes. Nevertheless, it results in different number of total GIMs x ESMs combinations among the indicators (e.g. 24 simulations for hydrology metrics against 12 simulations for agriculture indicators, Table 1).

**Table 1:** Summary of the ten analyzed climate-related multi-sectoral indicators from ISIMIP2b under the RCP6.0 scenario, with corresponding multi-model simulations, and stressors vs beneficial changes meaning depending on each projected change sign.

Sector	Yearly indicators	Stressors	Beneficial changes	Multi-model climate simulations	Impact model name
EXTREME	Very heavy rainy days	Increase	Decline	4 ESMs (4 simulations)	GFDL-ESM2M
WEATHER	Maximum consecutive dry days	Increase	Decline	//	HadGEM2-ES
	Highest heat stress value	Increase	Decline	//	IPSL-CM5A-LR
					MIROC5
HYDROLOGY	Highest runoff value	Increase	Decline	6 GIMs x 4 ESMs (24)	H08
	Lowest runoff value	Decline	Increase	//	LPJML
					MATSIRO
					ORCHIDEE
					PCR-GLOWB
					WATERGAP2
					GFDL-ESM2M
					HadGEM2-ES
					IPSL-CM5A-LR
					MIROC5
AGRICULTURE	Maize yields	Decline	Increase	3 GIMs x 4 ESMs (12)	GEPIC
	Wheat yields	Decline	Increase	//	LPJML
	Soy yields	Decline	Increase	//	PEPIC
	Rice yields	Decline	Increase	//	GFDL-ESM2M
					HadGEM2-ES
					IPSL-CM5A-LR
					MIROC5
FIRE	Burnt area fraction	Increase	Decline	4 GIMs x 4 ESMs (16)	CARAIB
					LPJ-GUESS
					LPJML
					VISIT
					GFDL-ESM2M
					HadGEM2-ES
					IPSL-CM5A-LR
					MIROC5

This table summarises the multi-sectoral indicators and their features, i.e. each stressor (adverse change) vs beneficial changes change sign and total Global Impact Models (GIMs) x Earth System Models (ESMs) simulations. Each GIM description can be found in [29] and each ESM description in [17]. All indicators are analysed as yearly values. Detailed descriptions of indicators are provided in Supporting Information S1 Text. **1.** Very heavy rainy days is the annual number of days with daily rainfall ≥ 20mm in days/year. **2.** Maximum consecutive dry days is the maximum number of consecutive days with daily rainfall ≤ 3mm in days/year. **3.** Highest extreme heat stress is the annual mean highest 2% daily simplified Wet-Bulb Globe Temperature (sWBGT) values with no unit. **4.** Highest runoff value is the annual mean highest 2% daily runoff values in mm/day. **5.** Lowest runoff value is the annual mean lowest 2% daily runoff values in mm/day. **6.** Maize yields in tons/hectare/year. **7.** Wheat yields in tons/hectare/year. **8.** Soy yields in tons/hectare/year. **9.** Rice yields in tons/hectare/year. **10.** Burnt area fraction in % of grid cell.

ISIMIP2b climate input data are restricted to four Earth System Models (ESMs) from CMIP5 [17] : two ESMs with higher climate sensitivity (i.e. IPSL-CM5A-LR and HadGEM2-ES) and two models with lower climate sensitivity (i.e. MIROC5, GFDL-ESM2M). This ESMs selection was heavily constrained by CMIP5 data availability [29]. In ISIMIP2b, climate input data are also restricted to two main Representative Concentration Pathways (RCPs) [37, 38], i.e. the low mitigated greenhouse gas emissions RCP6.0 scenario leading to a radiative forcing of +6 W/m2 in 2100, and the low emissions RCP2.6 leading to a radiative forcing of +2.6 W/m2 (note that the high emissions scenario RCP8.5 was added to the protocol at a later stage but with substantial fewer simulations). In this work we focus on the RCP6.0 scenario as it provides future simulations with a strong enough future climate change signal to detect possible TOE but it is not debated as a non-plausible future scenario [35, 36]). RCP6.0 also provides the highest number of multi-model simulations compared to RCP8.5, which allows quantifications of multi-model uncertainties and strengtens the robustness of this study.

### Time of Emergence (TOE)

We quantify the Time of Emergence (TOE) by defining the climate change signal within the 2006–2099 period, and the natural variability in the reference 1979–2005 period. Climate change and reference periods are chosen to separate the climate response to a future emission scenario (data from the RCP6.0 experiment) from the climate response to observed natural and anthropic forcings during the 20th century (data from the historical experiment [20]).

To detect those emergences, we use the Kolmogorov-Smirnov statistical test (KS-test) at 95% level of confidence [15, 39]. For each sectoral indicator, all 21-year windows distributions sliding from 2006 to 2089 are compared to the corresponding distribution over the 1985-2005 reference period: TOE is when the first sliding distribution is significantly different from the reference period. Altough the KS-test can be performed over distributions of various sizes (i.e. additional observations in one of the two samples), it does weaken the ability of the test to reject the null hypothesis when it is false [21]. For this reason, we use consistent 21-y windows both for sliding distributions and for the reference.

For each grid point, the KS-test checks whether the moving projected distribution is significantly different from the base period. When it is the case, the signal is considered to have emerged, and the moment of such signal is called the Time of Emergence TOE. The KS-test is sensitive to mean changes, but less sensitive to changes in the tails of the distribution [20]. Thus, if a TOE is detected for a given indicator, we consider this result only if the emerging change persists at least 20 years after the first detected TOE. As TOE detection can be sensisitive to different distribution sizes, we have also tested corresponding TOE calculations using 15-year and 25-year time windows. Results display very similar TOE results per indicator compared to the 21-year chosen window (not shown here).

### Signs of projected modification and related adverse vs beneficial future changes

In this paper, we analyse 10 climate-related multi-sectoral indicators based on previous multi-sectoral studies [24–27, 40] that illustrate climate impacts on extreme weather, hydrology, agriculture, and fire. Each sectoral indicator is detailed and described in Supporting information S1 Text.

Those indicators illustrate climate-related major sectors that may affect directly and indirectly people and societies. However, for each of those 10 analyzed sectoral indicators, a projected decreasing or an increasing trend can whether correspond to an adverse simulated change (a stressor) or a positive change (a benefit) for local population. For instance, a projected increase of the maximum consecutive dry days number per year is here considered as a stressor (i.e. higher risks of drought), whereas dry days number decline corresponds to a beneficial change (less drought risks) in our paper. Therefore, we define a stressor and a benefit for each of the 10 multi-sectoral indicators depending on its projected change sign (Table 1). We further assess those stressors and beneficial changes evolution and significance, and we describe emergences of cumulated cross-sectoral changes through TOE quantifications.

## Results

### Multi-model sectoral Times of Emergence (TOE)

**Fig 1:**
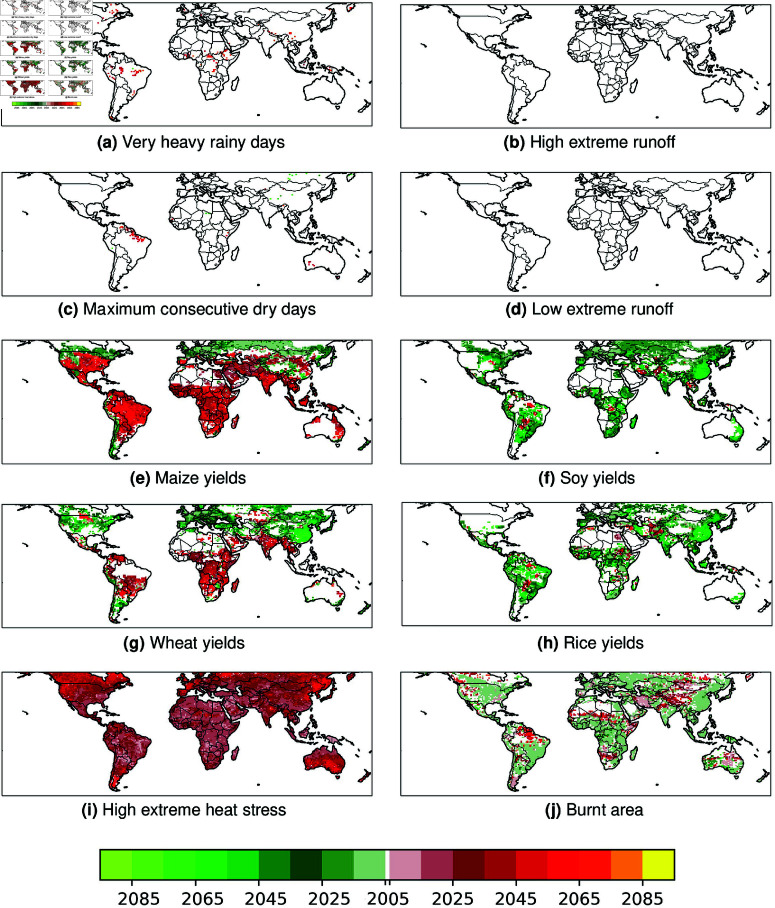
Spatial distributions of the multi-model median Time of Emergence (TOE) for the 10 analyzed climate-related multi-sectoral annual indicators under the RCP6.0 scenario. Corresponding future projected changes are provided in S1 Fig. **(a)** Number of days with daily precipitation *ge* 20mm (in days/year). **(b)** High extreme total runoff. **(c)** Maximum number of consecutives days with daily rainfall *le* 3 mm. **(d)** Low extreme total runoff. **(e)** Maize yields. **(f)** Soy yields/ **(g)** Wheat yields. **(h)** Rice yields. **(i)** High extreme heat stress (simplified Wet-Bulb Globe Temperature sWBGT). **(j)** Burnt area fraction. Per indicator, red colors illustrate a TOE of projected adverse changes (stressors), whereas green colors indicate a TOE of projected beneficial changes (benefits). White color indicates no TOE (no indicator value or detected TOE).

The global spatial distribution of multi-model median times of emergence (TOE) of climate related impacts under the RCP6.0 scenario shows various spatial patterns amongst the 10 selected climate-related cross-sectoral indicators (Fig 1). As explained in previous sections, a change for a given indicator can whether be a positive/beneficial change or a negative/adverse change (i.e. here after defined as a “stressor”) depending on its sign (Table 1). All along this paper and for all of the 10 indicators, beneficial changes include: less very heavy rainy days, less maximum consecutive dry days, lower annual extreme heat stress, lower highest runoff value, higher lowest runoff value, more yields of maize soy rice and wheat, and higher leaf area index. At the opposite, simulated stressors include: more very heavy rainy days, more maximum consecutive dry days, higher extreme heat stress, lower highest runoff value, lower lowest runoff value, less yields of maize soy rice and wheat, and lower leaf area index (Table 1).

Future multi-model median TOE is detected at a global scale mostly for 6 over the 10 analysed cross-sectoral climate indicators in ISIMIP2b simulations under the RCP6.0 scenario (1), and include emergences in: maize yields, soy yields, wheat yields, rice yields, high extreme heat stress and burnt area (Fig 1e–1j). Oppositively, very heavy rainy days and maximum consecutive dry days TOEs are sparsed and mostly detected for stressors in the tropics (Fig 1a, 1c), and no significant TOE is detected for high and low extreme runoffs (Fig 1b, 1d). Due to the selected MIRCA2000 harvested mask to relevantly represent and analyse possible modifications in crop yields [41], no crop yields TOE is detected over large parts of lands (Fig 1e–1h).

Most of detected cross-sectoral emergences are early during the 21st century (before 2020) at a global scale, both for beneficial and adverse projected changes (dark green and dark red colors in Fig 1 respectively). Specific location/indicator/change sign combinations are characterized by later TOE, for instance for detected TOE of beneficial changes in soy, rice and wheat yields in China, TOE of increasing extreme heat stress in northern high-latitudes or for TOE in decreasing maize yields in Amazonie (Fig 1). We provide a specific and detailed per sectoral indicator description of multi-model TOE for positive and adverse future changes in Supporting Information S2 Text.

### Cumulated TOE of climate-related cross-sectoral adverse changes (stressors) and beneficial changes (benefits)

**Fig 2:**
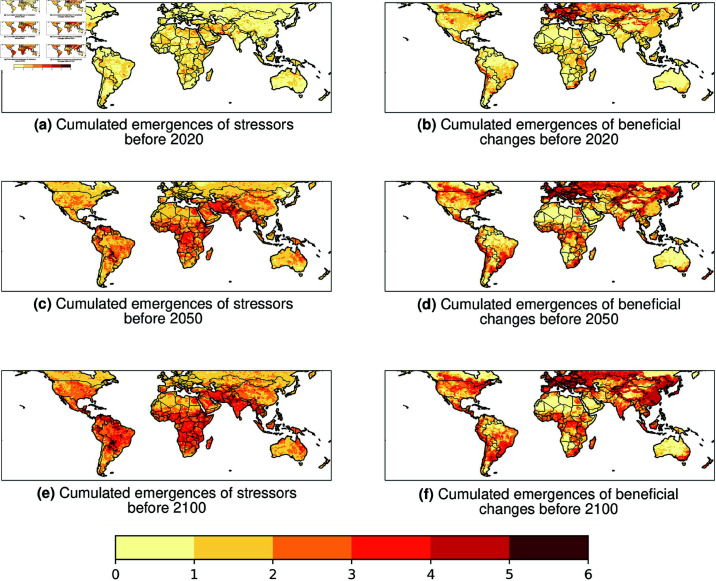
Spatial distributions of cumulated multi-model median emergences of the 10 selected climate-related cross-sectoral indicators. Left panels exhibit cumulated adverse TOE and right panels show cumulated beneficial TOE. Cumulated TOE are displayed as the sum of indicators from 0 to 10 that are chracterized by a detected multi-model median TOE before 2020 (top panels), before 2050 (middle panels), and before 2100 (bottom panels). Consistently with Fig 1, the colorbar has been adjusted from 0 to 6 (not 10), as almost no TOE is detected for 5 over the 10 analysed indicators.

To illustrate how many of the 10 selected climate-related cross-sectoral indicators are simulated to emerge and how these cross-sectoral emergences may spatially cumulate, we analyse the spatial distributions of cross-sectoral aggregated TOE both for adverse and beneficial projected changes under the RCP6.0 scenario (Fig 2).

Before 2020, almost no cumulated significant TOE is detected for projected adverse changes (Fig 2a, whereas a pattern of at least 4 cumulated emergences of beneficial changes is found in eastern Europe, southern Russia and sparsed regions of northern America (Fig 2b). These early cumulated beneficial emergences mostly include TOE detected for more crop yields and less burnt areas (Fig 1). Before 2050, large parts of the tropics including Central and East Africa, south-western Asia, and sparsed regions in northern South America are characterized by 4 to 6 cumulated adverse changes detected TOE (Fig 2c). These patterns are mainly driven by TOE detected for regional-scale projected decreasing maize yields, increasing extreme heat stress, increasing burnt area, and locally decreasing soy yields, wheat yields, and rice yields (Fig 1, S1 Fig). For beneficial changes, already emerging regions are characterized by 1 to 2 more detected TOE compared to before 2020, mostly driven by later local beneficial emergences in crop yields increases (Fig 1). By 2100, cumulated TOE patterns both for adverse and beneficial projected changes do not strongly differ from corresponding cumulated TOE patterns in 2050 (Fig 2e and 2f respectively). Patterns with maximum cumulated TOE in 2100 exhibit values of 6-7 in the tropics for adverse changes and in northern mid-latitudes for beneficial changes respectively, consistently with previous Fig 1 (and almost no TOE detected for extreme runoffs, very heavy rainy days and maximum consecutive dry days). This indicates that most of the detected TOE of cross-sectoral future changes (that is mostly 6 over 10 cross-sectoral indicators analysed) will all have emerged before 2050 according to ISIMIP2b and the RCP6.0 scenario.

Consistently with previous papers on future projected evolutions of multiple global warming cross-sectoral impacts [25–27], a dipole between adverse and beneficial aggregated emergences can be drawn between northern mid-latitudes and the tropics. Tropical regions here can be considered as a hotspot of cumulated emergent stressors from continuous global warming, whereas northern mid-latitudes can be characterized as a hotspot of *cumulated beneficial changes*. All these cross-sectoral stressors and benefits are mostly projected to emerge before 2050, with larger differences in the emergences amount between 2020 and 2050 emergences than between 2050 and 2100 (Fig 2).

### Earliest vs latest cross-sectoral TOE of stressors and beneficial changes

We analyse the earliest vs the latest cumulated cross-sectoral TOE per grid point for multi-model projected changes in RCP6.0, in order to better assess the time span of all cross-sectoral and aggregated emergences both for adverse and beneficial changes.

**Fig 3:**
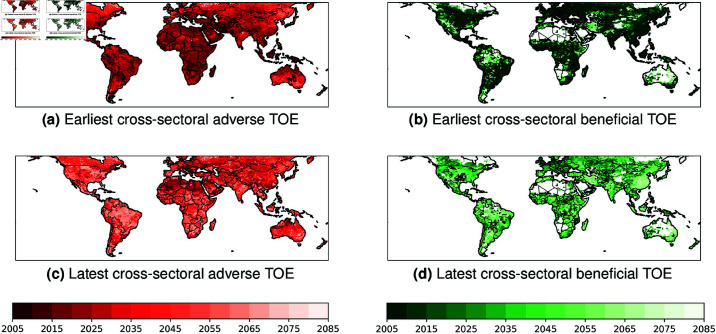
Spatial distributions of multi-indicator the earliest and the latest emergences for stressors and benefits. Values are obtained per grid point as the earliest and latest TOE during 2006-2100 among the 10 sectoral indicators.

The earliest cross-sectoral TOE (i.e. the earliest TOE among the 10 indicators analysed) is smaller than 2020 at a global scale both for future projected adverse and beneficial changes (Fig 3a and 3b), but with a particular early earliest cross-sectoral emergences for beneficial changes (i.e. TOE *leq* 2010). It indicates that earliest cumulated cross-sectoral stressors and benefits have globally already emerged according to ISIMIP2b simulations. However, adverse changes can be locally characterized by later earliest TOE, particularly in northern latitudes mid- and high- latitudes and in southern Australia (Fig 3a).

The latest cross-sectoral emergences show higher spatial and timing heterogeneities compared to earliest adverse and beneficial TOE (Fig 3c and Fig 3d respectively). Most lands are characterized by latest cross-sectoral TOE of stressors after 2050, with local latest TOE after 2060 (e.g. Amazonia, large parts of Africa). However, few regions including northern Africa, Arabia, large parts of ASIA and northern Australia exhibit that the latest adverse cross-sectoral emergence is detected before 2025 (Fig 3c). For beneficial future changes, the latest cross-sectoral TOE exhibit a higher spatial discrepandy compared to adverse changes (Fig 3d). A particular early latest cross-sectoral emergences is also shown over Europe (i.e. TOE *leq* 2020) and locally (e.g. central USA, eastern Africa, eastern Arabia).

Fig 3 here emphasizes the dipole between most of tropical regions and northern mid- and high-latitudes. According to ISIMIP2b, tropical regions are characterized by a long time-span of cross-sectoral emergences of stressors (i.e. 30 to 50 years), except northern Africa and Australia. Northern latitudes are however projected to experience almost “compound” emergences (≤ 10 years) of such stressors. For beneficial changes, regions with long (20 to 50 years) time-span of cross-sectoral emergences -including parts of northern America, south-east of South America, central Africa, many parts of Asia, eastern China- can be opposed to regions with almost no delay between earliest and latest cross-sectoral beneficial emergences (e.g. northern Canada, northern South America, northern Africa and Arabia, souther-western Africa, Australia).

### Multi-model TOE uncertainties at regional scales

**Fig 4:**
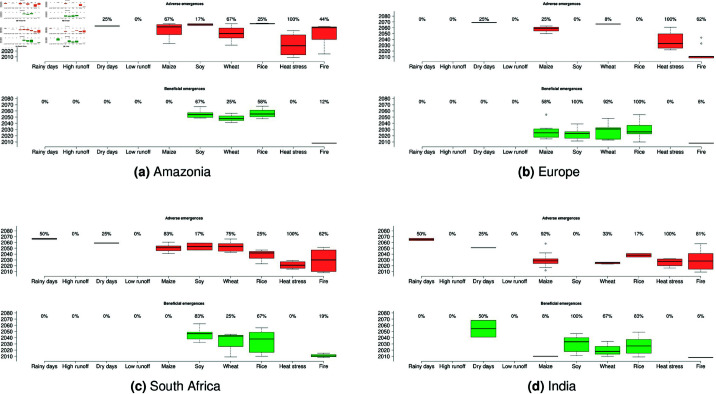
Boxplots illustrating regional multi-model spread in detected TOE for stressors (orange) and benefits (green) for four key regions. Percentages above each boxplot indicate models ratio exhibiting the corresponding spread/boxplot. Solid black lines display the multi-model median of regional mean TOE.

Most results presented above are based on the spatial distribution of multi-model median TOE of all ESMs x GIMs simulations detecting a TOE if at least 50% of the models agree on the sign of the indicator future change. However, and as shown in previous litterature, multi-sectoral and climate-related impacts projections come with large uncertainties, due to what can be named “the cascade of climate models, impact models, and cumulative approach uncertainties” [25]. In our study, the TOE quantification and related multi-model and multi-indicator uncertainties can be implemented to this “cascade”. Here we explore such uncertainties in multi-model regional cross-sectoral detected TOE for adverse and beneficial changes. For each indicator, the averaged regional TOE is calculated for each ESM x GIM combination, and all values are then displayed as a TOEs boxplot per indicator with its corresponding total multi-model detected TOE per change sign (Fig 4). We analyse these multi-model uncertainties for four regions : Amazonia, Europe, south of Africa and India.

All regions but Europe exhibit at least 7 indicators with a detected adverse emergence and a multi-model agreement (Fig 4). Indeed, Amazonia shows at least 50% of the ESM x GIM simulations agree on detected emergences of increasing dry days (50% of the simulations), maize and wheat yields declines (67%) and increasing heat stress (100%). No detected TOE nor multi-model agreement above 50% is found for other possible adverse emergences (Fig 4a). However, a TOE of beneficial change in soy and rice (i.e. more yields) is detected for 67 and 58% of the simulations respectively. South of Africa exhibit similar results, with mostly adverse changes emergences detected under a strong multi-model agreement (Fig 4c). Robustness is even larger for maize decline (83%), wheat decline (75%) and intensifying fires (62%). As for Amazonia, simulations also agree on the emergences of projected increasing soy and rice yields (83 and 67% respectively). India is characterized by a multi-model agreement of adverse emergences detected for rainy days (50 %), maize yields (92%), heat stress (100%) and fires (81%). For this region, simulations mostly agree on emergences of beneficial changes in dry days, soy, wheat and rice yields (Fig 4d). In Europe, only heat stress and fire indicators are characterized by robust multi-model averaged emergences of adverse changes (Fig 1b). Nevertheless, all crops are characterized by robust multi-model emergences of beneficial changes (58% agree for maize yields increase, 100% for soy, 92% for wheat and 100% for rice).

This uncertainties assessment strenghtens previous results and studies about aggregated multi-sectoral indicators [40]. We emphasize than for a same given indicator, strongly different TOE in sign of the trend and timing can be detected depending on each ESMs x GIMs simulations in ISIMIP2b (e.g. in South Africa for rice, 25% of the simulations detect an adverse TOE whereas 67% detect a beneficial TOE). Here we show that uncertain detection of TOE adds to the already considered *cascade of uncertainties* including uncertainties in climate models [42, 43], impact models [29], and sectoral indicators calculations [24–26, 40].

## Discussion

In this paper, multi-model spread and both Earth System Models (ESMs) and Global Impact Models (GIMs) uncertainties are accounted for each sectoral indicator. Previous papers have shown that most of the GIMs underestimate the extremeness of impacts in agriculture, terrestrial ecosystems, and heat-related mortality, while impacts on water resources are mainly overestimated with a large spread among the models [29, 44]. These multi-model uncertainties must be taken into account when considering our conclusions about the emergence of cumulated cross-sectoral adverse and beneficial changes.

To detect cross-sectoral TOE, we use the “Kolgomorov-Smirnov test” (KS-test) to statistically capture emergences from a prior variability. However, TOE can be detected using multiple statistical approaches [15, 23, 45, 46], and spatial distributions and mean TOE can thus vary depending on the selected indicator and region. Morevover, we set the first possible TOE detectable year at 2006, though previous papers have shown earlier TOE for different temperature and precipitation indicators during the historical period [15, 46]. Therefore, uncertainties in ISIMIP2b, aggregated impacts and TOE calculations here provide an *as robust as we can* framework and results of cross-sectoral emergences.

This paper accounts for upcoming emergences under the future RCP6.0 scenario, though both more emissive (RCP8.5) and less emissive (e.g. RCP2.6) future climate projections may result in different cross-sectoral TOE results. For instance, future projections show that maize yields will be characterized by emergent negative changes among major producer regions, with a TOE in 2032 (2051) under the highest (lowest) emissive scenario [23]. Nevertheless, as our major results emphasize emergences before 2050 for many cross-sectoral changes at regional scales, we argue that detected TOE both for stressors and benefits presented here are significant since corresponding RCPs in CMIP5 and ISIMIP2b follow similar emissive trajectories until 2040-2050 [37, 47].

In the present study, we consider all of the 10 indicators as independant, which may overestimate cumulated climate-related stressors and benefits [40, 48] and corresponding cumulated TOE. Multiple cross-sectoral seasonalities are also independently considered, although possible compound events and concurrent/synchronous occurrences might intensify adverse and beneficial emergences [49]. Present work does not quantify multivariate correlations e.g. temporal auto-correlations, space-time correlations or cross-correlations between indicators. Such statistics are important to depict accurate impact information from dynamical modelling [62, 63] and might modulate emphasized regime shifts. These statistical limitations must be accounted by the reader.

While this paper robustly depicts how multi-sectoral changes cumulatively emerge, it does not investigate underlying causes and mechanisms. Many studies have explored root causes behind observed and simulated changes per individual sector, but no paper have analysed processes behind emergences of multi-sectoral cumulated changes to our knowledge. For instance, projected changes in crop yields can be mostly reconstructed based on shifts in global mean temperature at a global scale [3, 5], but changes in peak runoff and related causes differ depending on whether simulations come from biome models (e.g. LPJML, VISIT) or hydrological models (e.g. H08, PCR-GLOWB, WATERGAP2) [50]. Few papers have only demonstrated that the assessment of processes explaining cumulated changes is not trivial. For instance, small concordances between spatial patterns of changes in flood/drought risks and precipitation were shown, although rainfall is an underlying driver of both risks [27]. Further work about these underlying causes of cross-sectoral cumulated patterns and their time constraints would significantly improve our ability to anticipate additive changes in various sectors. This would further help anticipating adapted copying strategies both per sector and at a multi-/cross- sectoral level [51].

Here we provide a multi-model cross-sectoral assessment of future projected changes in terms of “stressors” and “beneficial changes”. Nevertheless, we do not quantify related vulnerability nor exposure to translate such stressor vs beneficial change insights into a proper risk vs benefits information. As specified in the IPCC, both definition and level of resulting risks (and allegedly benefits) result both from changes magnitude but also from how population and its regional behaviours evolve [58]. As an example, scenarios with higher demand for water, feed, and/or with high consumption of resources and limited technological improvements induce higher risks, and such future evolution may significantly modulate our adverse vs positive changes findings in terms of related risks vs benefits from global warming impacts.

Previous papers have shown that climate models with similar global mean warming can lead to different aggregated impacts, so that climate model uncertainties remain a significant contributor to sectoral and cross-sectoral analysis uncertainties [44]. Though out of the scope of this paper, a further step could be to investigate these uncertainties and assess ESMs × GIMs simulations contribution to the total multi-model TOE uncertainty both per indicator and in total cumulated stressors (benefits). It would also provide an assessment about which ESMs and GIMs and their combinations agree better or are out of the multi-model range, as done previously for cumulated impacts changes [49, 52, 53].

ISIMIP dataset provides multi-ensemble robust projections of various sectoral evolutions that incorporates a wide range of climate and impact models [29]. This dataset allows the identification of regional disparities, which may help the development of region-specific policies and adaptation strategies in further studies. Indeed, ISIMIP can provide valuable insights about the interlinkages of climate impacts and their implications for disaster risk management [54]. By integrating such related ISIMIP key findings, policymakers could develop sectoral prioritized strategies to enhance resilience regional/local, improve early warning systems, or effectively allocate resources to mitigate adverse impacts of climate-related disasters. Nevertheless, ISIMIP limitations should be considered due to corresponding needed availability and quality of input data. These uncertainties might result in a low confidence in sectoral simulations over regions where data is limited [55]. Furthermore, the multi-model approach performed in ISIMIP involve often-poorly simulated extremes [60, 61]. Particularly, multiplicative bias correction used in ISIMIP protocols improve mean statistic but may worsen related extremes statistics such as variance [59]. Some interpolation techniques such as “nearest neighbor” might also dampen the end tail of intensity distributions. As we particularly focus on what may be experienced by people in terms of emergences of “stressors” and beneficial changes, those limitations in extreme representations must be kept in mind by the reader when interpreting our key findings [61].

At the time of this manuscript writting, the ISIMIP2b phase using CMIP5 climate input was the most recent completed multi-sectoral ISIMIP version needed for our cross-sectoral approach. However, the recent ISIMIP phase 3 and its datasets are derived from the most recent CMIP6 multi-model climate simulations with improved bias-adjustment and downscaling methods. Sectoral specific studies have already exhibited that CMIP6 multi-model crop projections show more pronounced impacts of climate change on global crop yields compared to CMIP5 [23]. Those studies suggest that our present multiple impacts TOE assessment but using ISIMIP3/CMIP6 projections would exhibit i) stronger cumulated adverse and beneficial cross-sectoral future changes, ii) possibly earlier TOE both for adverse and beneficial changes.

Such an assessment of the timing of projected various changes for different climate-related sectors is crucial for defining priorities for adaptation and provides evidence of the risks led by global warming. Related risks are context-specific and are observed and projected to be higher in regions that are already experiencing severe weather conditions, with limited institutional and socio-economic resources for adaptation [56]. Consequent cumulated risks for people’s lives can be accordingly amplified and severe, and may include-but are not limited to- regional decline in water supply and quality, increasing risk of disease and mental health issues, more displacements and migrations, economic and income losses, declining infracstructures (transport, electricity) [27]. Accelerating global warming is also increasing climate impacts and puts countries at serious risk of experiencing severe losses and damages. To face such expected climate-related changes, global efforts in adaptation financing and planning continue to make incremental progress at national and international levels [30], with current actions concentrated in sectors of water, agriculture and ecosystems, and primarily addressing rainfall variability, flood and drought risks. Nevertheless, the global warming acceleration results in increasing risk of adaptation limits, and global efforts fail to keep pace with such adverse changes [31]. Our cross-sectoral emergences study here feeds the scientific basis to help foresee possible strong climate changes and plan adaptation and mitigation strategies. This paper provides reliable time constrains about multiple adverse and beneficial climate-related shifts in sectors that will particularly affect populations and societies in the upcoming decades.

## Conclusion

This paper aims to emphasize for the first time how multi-sectoral impacts of global warming emerge from the natural variability, and how emergences could occur and cumulate in sectors which strongly affect populations (e.g. agriculture, hydrology, extreme weather). To do so, we quantify the Time of Emergence (TOE) of both adverse and beneficial simulated changes in 10 multi-sectoral indicators using ISIMIP2b simulations under the RCP6.0 future scenario.

Major results show that:

- Over the 10 multi-sectoral indicators assessed, multi-model averaged TOE is largely detected at a global scale for 6 of them. Most of these emergences are characterized by a multi-model mean TOE earlier than 2015, illustrating an already emerged new climate regime for various sectors (i.e. agriculture, hydrology, weather extremes). However, these cross-sectoral emergences do not necessarily occur at the same time at local levels. For instance, spatial distributions of multi-model mean TOE exhibit an emergence before 2015 for future maize yields increase in northern latitudes, but a TOE after 2050 for increasing wheat yields in eastern China.
- TOE of such metrics are detected both for adverse and beneficial projected changes at the global scale. According to ISIMIP2b, northern mid- and high- latitudes will mostly experience cumulated positive cross-sectoral emergences (up to 6 benefits), whereas the tropics will experience multiple adverse emergences (4 to 6 stressors).
- Most of the cumulated cross-sectoral TOE over the 21st century are detected before 2050 under the RCP6.0 scenario, with more TOE of changes detected over the 2006-2050 period than over 2050-2100. Cumulated beneficial changes are simulated in northern latitudes to have already emerged (before 2020), whereas most cross-sectoral adverse emergences are simulated in the tropics to emerge before 2050.
- For all sectoral metrics but annual extreme heat stress and crop yields in northern latitudes, substantial multi-model cross-sectoral TOE uncertainties are exhibited. Consistently with previous papers about aggregated multi-sectoral indicators, we emphasize that TOE uncertainties adds to the already considered *cascade of uncertainties* from impact models, climate models, and sectoral indicators selection and calculations.

This paper presents a comprehensive multi-model assessment of the timing and contextual dynamics of cumulative multi-sectoral climate-related adverse and beneficial changes in the upcoming decades. However, it is important to acknowledge inherent limitations coming with such approaches, including large uncertainties in impact models and Earth System Models (ESMs), the use of a single future greenhouse gases scenario, and independantly considered stressors/benefits among sectors. Additionally, the study draws upon prior CMIP-based simulations, adding a layer of uncertainties but providing perspectives for future work in order to strenghten our findings at the global scale. Further studies could focus on the severity of each individual sector regarding the overall emergent patterns of adverse and beneficial changes. Evidence of cross-sectoral hierarchical “worst to better” [40] of such stressors/risks would also significantly help priorizing region-specific adaptation strategies.

Present identified early and cumulative cross-sectoral adverse emergences highlight the urgent need for effective mitigation and adaptation strategies. Given the specific vulnerabilities and risks faced by tropical regions, policies should be tailored to address correponding unique challenges [57]. This accordingly includes targeted support for agricultural practices that are resilient to climate change, improved water resource management systems, and investment in early warning systems for extreme weather events. In this context, future studies should focus on improving climate models, impacts models, and the aggregation of cross-sectoral impacts to reduce uncertainties associated with the detection of TOE. Such detected emergence signals from impact simulations, particularly early 2010s–2020s ones, could be also compared to possibly detected TOE from related observations, despite the current lack of post-2010s observations to robustly assess the persistence of recent emergences. This further work could involve refining models to better capture regional dynamics, integrating observational data with modeling approaches, and increasing the resolution and accuracy of projections (e.g. CMIP6, ISIMIP3).

## Supporting information

S1 Text Methods: Indicators descriptions. Per indicator description (literature and model features) of all of the 10 multi-sectoral climate-related indicators
(PDF)

S2 Text Results: Global scale TOE description for each indicator according to ISIMIP2b under the RCP6.0 scenario (related to main paper Fig 1).
(PDF)

S1 Fig Multi-model future projected changes in the 10 indicators according to ISIMIP2b under the RCP6.0 scenario. (PDF)

S2 Fig Spatial distributions of multi-model median projected stressors TOE for the 10 indicators. (PDF)

S3 Fig Spatial distributions of multi-model median projected benefits TOE for the 10 indicators. (PDF)
