# Supplementary material for: Multiple climate-related stressors in the tropics and beneficial changes in northern latitudes will mostly have emerged before 2050
Source: PLoS One. 2025 Jun 17;20(6):e0293551. doi: 10.1371/journal.pone.0293551 (PMC12173232; doi:10.1371/journal.pone.0293551)
Supplement: S1 Text — Per indicator description (literature and model features) of all of the 10 multi-sectoral climate-related indicators (PDF) [file pone.0293551.s001.pdf]

TEXT S1 - Methods: Indicators descriptions

In the present study, we fairly represent major sectors affecting populations and societies according to litterature including water ressourches, agriculture, climate extremes and fire[1–5]. The following section details indicators we select to provide an accurate analysis about multiple and cumulated emergences of cross-sectoral risks and benefits at global scale.

Drought risks

Hotter and dryer hotter conditions intensify water needs of organisms and population, particularly in regions already water stressed as the tropics. Regionally, water scarcity results from a complex feedback and equilibrium between extreme hot temperatures, lack of precipitation, and lack of soil moisture[6]. Drought metrics were thus developped for land-atmosphere feedbacks and physical understanding, as well as for impact analyses. In previous works, the magnitude of the 5% lowest daily runoff per year (values $\leq$ 5th percentile) were defined as an indicator of low flow[5,7]. According to ISIMIP2b data availability ([8]), we similarly analyse the yearly lowest 2% daily runoff of each year (values $\leq$ the 2th percentile), and here consider this metric as a *low flow* index and thus a drought risk indicator. In addition, we quantify the number of consecutive days with daily precipitation smaller than 3mm per year (here after *consecutive dry days*), to strenghten and compare results of drought metrics[5,9].

Flood risks

Flood data strongly depends on the available data, which depend on rainfall-runoff modelling, regional flood statistics, and local flood statistics[10–12]. Flood studies usually require data of river catchment, rivers, and case study of real flood events. These data are then compared to flood model data to investigate needed conditions to exhibit possible flood event depending on regional conditions. Due to ISIMIP2b data availability and with the purpose to analyse the highest number of combined GIMs $\times$ ESMs simulations, here we assess the highest daily runoff per year as a flood risk index similarly as in [Brouillet and Sultan 2023][5] As for droughts, previous litterature defines the highest 5% daily runoff values per year as an indicator of yearly *high flow*[7]. Similarly, we thus quantify the annual highest 2% daily runoff (values $\geq$ 98th percentile) as an *high flow* index and thus a flood risk indicator. In addition, we quantify the number of days per year when daily precipitation are higher than 20mm, referenced here after as a *very heavy rainy days* indicator[9].

Agriculture

To illustrate simulated and future projected risks and benefits in the sector of agriculture, and according to available ISIMIP2b data, here we analyse simulated crop yields of the four major staple annual crop yields (maize, soybean, wheat and rice) as done in previous studies[13–16].

Heat stress : calculation of the simplified Wet-Bulb Globe Temperature (sWBGT)

Under very hot and humid conditions, the ability of the human body to regulate the core temperature and dissipate heat via sweat evaporation is reduced, provoking heat stress[17,18]. Such conditions strongly affect human well-being and can develop into heat-related illnesses such as fatigue, muscle cramps and heat stroke[19,20], as well as labor productivity decline[21,22]. In this context, many heat stress indices to study those heat-related risks have been developped[23]. In the present study we focus on the usually used simplified Wet-Bulb Global Temperature (sWBGT) to easily compare our results with actual litterature, and as this indicator only needs climate variables to be calculated following the equation :

$$sWBGT = (0.567 \times T) + (0.393 \times VP) + 3.94 \tag{1}$$

with sWBGT in no unit (but values close to °C), T the near-surface air temperature (in °C), and VP the air vapor pressure (in hPa). VP is calculated from the four CMIP5 ESMs in ISIMIP2b daily data of specific humidity (q), sea-level pressure (slp) and T as :

$$VP = \frac{q \times psurf}{0.622 + 0.378 \times q} \tag{2}$$

with q the surface specific humidity (in $kg_{water}/kg_{humid.air}$) and psurf the surface air pressure (in hPa). Psurf is not directly provided as daily simulations, and is derived from the sea-level pressure using hydrostatic equilibrium and assuming adiabatic conditions as:

$$psurf = psl \times \exp\left(\frac{-g \times z}{r \times T}\right) \tag{3}$$

with psurf and psl in hPa. g is the gravitational constant (9.81 m/s^{-2}), r the gaz constant of the air with a value of 287.067046 J/kg/K (obtained from dividing the molar gas constant R (i.e. 8.3144321 J/mol/K) with the molar mass of the air (i.e. 29 g/mol). T is the surface air temperature in K, and z the altitude in meters.

Fires

Wildfire impacts are known to be severe for population and include risks of asphyxiation, injuries, health issues from smoke exposure or drinkable water contamination, destroyed homes, agricultural land losses[24]. Like climate and vegetation, fire activity and regimes can change over time in response to changing drivers, and interactions and dependencies between climate, fire, people and vegetation are still being unravelled[25,26]. Burned area data available in ISIMIP2b consistently provide a consistent global perspective on changing patterns of fire activity both at actual and future simulated scales. Here we aggregate monthly burnt area data into annual averages in order to provide robust multi-model evolutions of how wildfire activity is projected to evolve and how significant changes may emergences both for adverse and beneficial changes.

References

1. Piontek, F. *et al.* Multisectoral climate impact hotspots in a warming world. *Proc. Natl. Acad. Sci.* **111**, 3233–3238, DOI: 10.1073/pnas.1222471110 (2014).
2. Mora, C. *et al.* Global risk of deadly heat. *Nat. Clim. Chang.* **7**, 501–506, DOI: 10.1038/nclimate3322 (2017).
3. Byers, E. *et al.* Global exposure and vulnerability to multi-sector development and climate change hotspots. *Environ. Res. Lett.* **13**, 055012, DOI: 10.1088/1748-9326/aabf45 (2018).
4. Chen, Y., Moufouma-Okia, W., Masson-Delmotte, V., Zhai, P. & Pirani, A. Recent Progress and Emerging Topics on Weather and Climate Extremes Since the Fifth Assessment Report of the Intergovernmental Panel on Climate Change. *Annu. Rev. Environ. Resour.* **43**, 35–59, DOI: 10.1146/annurev-environ-102017-030052 (2018).
5. Brouillet, A. & Sultan, B. Livestock exposure to future cumulated climate-related stressors in west africa. *Sci. Reports* **13**, 2698, DOI: 10.1038/s41598-022-22544-y (2023).
6. Sylla, M. B., Gaye, A. T., Jenkins, G. S., Pal, J. S. & Giorgi, F. Consistency of projected drought over the Sahel with changes in the monsoon circulation and extremes in a regional climate model projections. *J. Geophys. Res. Atmospheres* **115**, DOI: 10.1029/2009JD012983 (2010).
7. Gosling, S. N. *et al.* A comparison of changes in river runoff from multiple global and catchment-scale hydrological models under global warming scenarios of 1 °C, 2 °C and 3 °C. *Clim. Chang.* **141**, 577–595, DOI: 10.1007/s10584-016-1773-3 (2017).
8. Davie, J. C. S. *et al.* Comparing projections of future changes in runoff from hydrological and biome models in ISI-MIP. *Earth Syst. Dyn.* **4**, 359–374, DOI: 10.5194/esd-4-359-2013 (2013).
9. Sillmann, J., Kharin, V. V., Zwiers, F. W., Zhang, X. & Bronaugh, D. Climate extremes indices in the CMIP5 multimodel ensemble: Part 2. Future climate projections: CMIP5 PROJECTIONS OF EXTREMES INDICES. *J. Geophys. Res. Atmospheres* **118**, 2473–2493, DOI: 10.1002/jgrd.50188 (2013).
10. Zehe, E., Elsenbeer, H., Lindenmaier, F., Schulz, K. & Blöschl, G. Patterns of predictability in hydrological threshold systems. *Water Resour. Res.* **43**, DOI: 10.1029/2006WR005589 (2007).
11. Blöschl, G. *et al.* At what scales do climate variability and land cover change impact on flooding and low flows? *Hydrol. Process.* **21**, 1241–1247, DOI: 10.1002/hyp.6669 (2007).
12. Arnell, N. W. & Lloyd-Hughes, B. The global-scale impacts of climate change on water resources and flooding under new climate and socio-economic scenarios. *Clim. Chang.* **122**, 127–140, DOI: 10.1007/s10584-013-0948-4 (2014).
13. Rosenzweig, C. *et al.* Assessing agricultural risks of climate change in the 21st century in a global gridded crop model intercomparison. *Proc. Natl. Acad. Sci.* **111**, 3268–3273, DOI: 10.1073/pnas.1222463110 (2014).
14. Ostberg, S., Schewe, J., Childers, K. & Frieler, K. Changes in crop yields and their variability at different levels of global warming. *Earth Syst. Dyn.* **9**, 479–496, DOI: 10.5194/esd-9-479-2018 (2018).
15. Müller, C. *et al.* Exploring uncertainties in global crop yield projections in a large ensemble of crop models and CMIP5 and CMIP6 climate scenarios. *Environ. Res. Lett.* DOI: 10.1088/1748-9326/abd8fc (2021).
16. Jägermeyr, J. *et al.* Climate impacts on global agriculture emerge earlier in new generation of climate and crop models. *Nat. Food* **2**, 873–885, DOI: 10.1038/s43016-021-00400-y (2021).
17. Parsons, K. Heat stress standard ISO 7243 and its global application. *Ind. health* **44**, 368–379 (2006).

18. Casanueva, A. *et al.* Climate projections of a multivariate heat stress index: the role of downscaling and bias correction. *Geosci. Model. Dev.* **12**, 3419–3438, DOI: 10.5194/gmd-12-3419-2019 (2019).

19. Kjellstrom, T. *et al.* Heat, Human Performance, and Occupational Health: A Key Issue for the Assessment of Global Climate Change Impacts. *Annu. Rev. Public Heal.* **37**, 97–112, DOI: 10.1146/annurev-publhealth-032315-021740 (2016).

20. Coccolo, S., Kämpf, J., Scartezzini, J.-L. & Pearlmutter, D. Outdoor human comfort and thermal stress: A comprehensive review on models and standards. *Urban Clim.* **18**, 33–57, DOI: 10.1016/j.uclim.2016.08.004 (2016).

21. Kjellstrom, T., Holmer, I. & Lemke, B. Workplace heat stress, health and productivity – an increasing challenge for low and middle-income countries during climate change. *Glob. Heal. Action* **2**, 2047, DOI: 10.3402/gha.v2i0.2047 (2009).

22. Dunne, J. P., Stouffer, R. J. & John, J. G. Reductions in labour capacity from heat stress under climate warming. *Nat. Clim. Chang.* **3**, 563–566, DOI: 10.1038/nclimate1827 (2013).

23. de Freitas, C. R. & Grigorieva, E. A. A comprehensive catalogue and classification of human thermal climate indices. *Int. J. Biometeorol.* **59**, 109–120, DOI: 10.1007/s00484-014-0819-3 (2015).

24. Mora, C. *et al.* Broad threat to humanity from cumulative climate hazards intensified by greenhouse gas emissions. *Nat. Clim. Chang.* **8**, 1062–1071, DOI: 10.1038/s41558-018-0315-6 (2018).

25. Archibald, S. Managing the human component of fire regimes: lessons from africa. *Philos. Transactions Royal Soc. B: Biol. Sci.* **371**, 20150346, DOI: 10.1098/rstb.2015.0346 (2016).

26. Andela, N. *et al.* A human-driven decline in global burned area. *Science* **356**, 1356–1362, DOI: 10.1126/science.aal4108 (2017).
