# Supplementary material for: Multiple climate-related stressors in the tropics and beneficial changes in northern latitudes will mostly have emerged before 2050
Source: PLoS One. 2025 Jun 17;20(6):e0293551. doi: 10.1371/journal.pone.0293551 (PMC12173232; doi:10.1371/journal.pone.0293551)
Supplement: S2 Text — according to ISIMIP2b under the RCP6.0 scenario (related to main paper Fig 1). (PDF) [file pone.0293551.s002.pdf]

TEXT S2 - Results: Global scale TOE description for each indicator

Extreme weather

For the very heavy rainy days indicator, almost no TOE is detected at a global scale, except over few located pixels (main Fig 1a). Within these few regions, TOE is mostly detected for projected increasing very heavy rainy days (i.e. stressor), particularly along the equatorial band and eastern Canada. This adverse change is simulated to emerge between around 2055 within the tropics along with a projected increase of 100% of the historical value by 2074-2100 (Figure S1). Shown projected changes in very heavy rainy days are consistent with current literature showing that heavy precipitation events will become more intense and more frequent with additional global warming[1]. At regional scales, the intensification of such extreme events will depend on the amount of regional warming and changes in atmospheric circulation and storm dynamics[2]. Detected TOE are also consistent with previous regional TOE assessment over West Africa that shows only sparse locations with TOE of increasing very wet days and large no-detected-TOE areas[3]. Such emergences seem to be explained by the limited multi-model robustness and the Kolmogorov-Smirnov test use. In our study, the limited number of total multi-model simulations for this indicator (i.e. 4 ESMs in ISIMIP2b) may largely contribute to the poor robustness that results in the non-detected multi-model median TOE exhibited at a global scale.

As for very heavy rainy days, no-detected-TOE areas dominate at global scale for projected changes in the maximum consecutive number of dry days (main Fig. 1c). In sparsed regions with detected TOE, multi-models show TOE of increasing dry days (stressor) in north-eastern Amazonia, Senegal, and western Australia, and TOE of decreasing maximum consecutive dry days in few pixels in Asia. Corresponding emergences are associated with a projected increase by 40-50% (adverse change) and a decrease by 20% (beneficial change) respectively (Fig S1).

Multi-model projections of annual extremes of simplified Wet-Bulb Globe Temperature (sWBGT, the heat stress index) show a significant intensification of heat stress extremes (stressor) over all land at global scales (Fig S1i), with corresponding TOE before 2010 and no beneficial change nor emergence (main Fig. 1i). This robust and global adverse emergence results from the temperature and warming effects that prevails on the humidity factor (global drying) in the sWBGT calculation and projected intensification[4–6]. This indicator also exhibits a better agreement due to a better multi-model agreement in future projections of temperature (Fig S1i) and related metrics than for rainfall extremes (Fig S1a and S1c). The slight later sWBGT TOE over northern mid-latitudes latitudes can be explained by the specific temperature increase of annual extreme sWBGT occurrence that warms faster in the tropics than within northern latitudes due to role of relative humidity future changes[6,7].

Hydrology

Both for high and low extreme runoff, no TOE is detected (main Fig. 1b and 1d). This no-detection is consistent with corresponding very small amount of detected TOE for very rainy days and maximum consecutive dry days indicators (main Fig. 1a and 1c). It is also consistent with known literature, since previous studies have shown there is only low multi-model confidence about peak flow trends in past decades observations and in future projections at global scales[1].

Without being able to detect any emergence for these runoff indicators, here we show consistent[8] future adverse changes in high extreme runoff (increase) over large parts of the tropics and Asia, and future beneficial changes in high runoff (decrease) mostly within the Mediterranean basin and Australia (Figure S1b). Accordingly, low extreme runoff exhibit future adverse projection in drying regions (USA, Europe and Mediterranean, Amazonia), and beneficial future changes in Sahel, eastern Africa and northern latitudes (Figure S1d). Theses drying vs wettening changes are consistent with previous papers about the expected intensifying drying trend over the mid-continental areas[6,9] and the projected wettening of some regions e.g. East Africa[10,11]. Despite such robust projected increasing and decreasing changes of high and low extreme runoff at a global scale, we do show no abrupt and/or persistent change in ISIMIP2b simulations for the RCP6.0 scenario for high and low extreme runoff.

Agriculture

TOE of crop yields show varying spatial patterns depending on the cereal and the region (main Fig. 1e-h). Maize yields are characterized by a future projected decline at a global scale, with related adverse emergence detected between 2030 and 2050. However, increasing maize yields are simulated over northern high- and mid-latitude with corresponding TOE before 2020 (Fig 1e, Fig S1e). Both soy and rice yields show almost only beneficial TOE at a global scale over corresponding current harvested crops ([12]) with earliest corresponding TOE in Europe and located regions in Africa (before 2010), and latest TOE over large parts of Amazonia and eastern China (main Fig. 1f-h, Figures S1f-h). As for maize, wheat projections show yields decline and associated adverse TOE of 2020-2040 over the extended tropics, but increasing yields (benefits) and emergences over a larger extend in northern mid-latitudes. This includes beneficial TOE detected between 2010 and 2060 over all northern America and large parts of Asia (main Fig. 1g, Figure S1g). Our findings are consistent with previous studies that have shown a negative global warming effect on crop yields in the tropics, stronger on maize and wheat compared to soy and rice productions, but a positive (beneficial) effect in higher and northern latitudes[13,14]. Zonal TOE differences are explained by the beneficial effects of the warming and the increasing CO2 fertilization in regions with moderate temperature conditions (i.e. northern latitudes)

and the strong adverse warming effect where regional climate is already stressful for suitable production such as in the tropics ([15]). Here we provide additionnal details about the timing of such trends, and we show that significant changes in the four major crop yields have already started to emerge because of global warming.

Fire

Most of lands are characterized by multi-model median beneifical and early emergences of burnt area changes (TOE $\leq$ 2015, main Fig. 1j). Oppositively, many located and sparsed regions are characterized by an emergence of adverse changes from 2035, including western North America, northern South America, Sahel, south of Africa, large parts of Asia and Australia.

When considering the robustness of multi-model future projected changes in burnt area fractions, regions with projected increasing fires (northern mid-latitudes and southern tropics) are characterized by at least 66% of the simulations agreeing, whereas other regions and particularly regions with detected beneficial early TOE show a poor multi-model agreement according to ISIMIP2b. These multi-model uncertainties both in detected changes and TOE may result from misrepresented and/or unrepresented direct human influences on wildfire management, ignition and suppression in corresponding vegetation models[16,17].

References

1. S.I., S. *et al.* Weather and climate extreme events in a changing climate. In *Climate Change 2021: The Physical Science Basis. Contribution of Working Group I to the Sixth Assessment Report of the Intergovernmental Panel on Climate Change [Masson-Delmotte, V., P. Zhai, A. Pirani, S.L. Connors, C. Péan, S. Berger, N. Caud, Y. Chen, L. Goldfarb, M.I. Gomis, M. Huang, K. Leitzell, E. Lonnoy, J.B.R. Matthews, T.K. Maycock, T. Waterfield, O. Yelekçi, R. Yu, and B. Zhou (eds.)].*, 1513–1766, DOI: doi:10.1017/9781009157896.013 (Cambridge University Press, 2021).
2. Fischer, E. M. & Knutti, R. Anthropogenic contribution to global occurrence of heavy-precipitation and high-temperature extremes. *Nat. Clim. Chang.* **5**, 560–564, DOI: 10.1038/nclimate2617 (2015).
3. Gaetani, M., Janicot, S., Vrac, M., Famien, A. M. & Sultan, B. Robust assessment of the time of emergence of precipitation change in West Africa. *Sci. Reports* **10**, 7670, DOI: 10.1038/s41598-020-63782-2 (2020).
4. Buzan, J. R., Oleson, K. & Huber, M. Implementation and comparison of a suite of heat stress metrics within the Community Land Model version 4.5. *Geosci. Model. Dev.* **8**, 151–170, DOI: 10.5194/gmd-8-151-2015 (2015).
5. Zhao, Y., Ducharne, A., Sultan, B., Braconnot, P. & Vautard, R. Estimating heat stress from climate-based indicators: present-day biases and future spreads in the CMIP5 global climate model ensemble. *Environ. Res. Lett.* **10**, 084013, DOI: 10.1088/1748-9326/10/8/084013 (2015).
6. Brouillet, A. & Joussaume, S. Investigating the Role of the Relative Humidity in the Co-Occurrence of Temperature and Heat Stress Extremes in CMIP5 Projections. *Geophys. Res. Lett.* **46**, 11435–11443, DOI: 10.1029/2019GL084156 (2019).
7. Brouillet, A. & Joussaume, S. More perceived but not faster evolution of heat stress than temperature extremes in the future. *Clim. Chang.* DOI: 10.1007/s10584-020-02752-z (2020).
8. Hirabayashi, Y. *et al.* Global flood risk under climate change. *Nat. Clim. Chang.* **3**, 816–821, DOI: 10.1038/nclimate1911 (2013).
9. Fischer, E. M. & Knutti, R. Robust projections of combined humidity and temperature extremes. *Nat. Clim. Chang.* **3**, 126–130, DOI: 10.1038/nclimate1682 (2013).
10. Nguyen, T.-H., Min, S.-K., Paik, S. & Lee, D. Time of emergence in regional precipitation changes: an updated assessment using the CMIP5 multi-model ensemble. *Clim. Dyn.* **51**, 3179–3193, DOI: 10.1007/s00382-018-4073-y (2018).
11. Ayugi, B. *et al.* Future Changes in Precipitation Extremes over East Africa Based on CMIP6 Models. *Water* **13**, 2358, DOI: 10.3390/w13172358 (2021).
12. Portmann, F. T., Siebert, S. & Döll, P. MIRCA2000-Global monthly irrigated and rainfed crop areas around the year 2000: A new high-resolution data set for agricultural and hydrological modeling: MONTHLY IRRIGATED AND RAINFED CROP AREAS. *Glob. Biogeochem. Cycles* **24**, n/a–n/a, DOI: 10.1029/2008GB003435 (2010).
13. Ostberg, S., Schewe, J., Childers, K. & Frieler, K. Changes in crop yields and their variability at different levels of global warming. *Earth Syst. Dyn.* **9**, 479–496, DOI: 10.5194/esd-9-479-2018 (2018).
14. Tigchelaar, M., Battisti, D. S., Naylor, R. L. & Ray, D. K. Future warming increases probability of globally synchronized maize production shocks. *Proc. Natl. Acad. Sci.* **115**, 6644–6649, DOI: 10.1073/pnas.1718031115 (2018).
15. Rosenzweig, C. *et al.* Assessing agricultural risks of climate change in the 21st century in a global gridded crop model intercomparison. *Proc. Natl. Acad. Sci.* **111**, 3268–3273, DOI: 10.1073/pnas.1222463110 (2014).

16. Andela, N. *et al.* A human-driven decline in global burned area. *Science* **356**, 1356–1362, DOI: 10.1126/science.aal4108 (2017).

17. Lange, S. *et al.* Projecting Exposure to Extreme Climate Impact Events Across Six Event Categories and Three Spatial Scales. *Earth's Futur.* **8**, e2020EF001616, DOI: https://doi.org/10.1029/2020EF001616 (2020).
