# Supplementary material for: Multiple climate-related stressors in the tropics and beneficial changes in northern latitudes will mostly have emerged before 2050
Source: PLoS One. 2025 Jun 17;20(6):e0293551. doi: 10.1371/journal.pone.0293551 (PMC12173232; doi:10.1371/journal.pone.0293551)
Supplement: S1 Fig — (PDF) [file pone.0293551.s003.pdf]

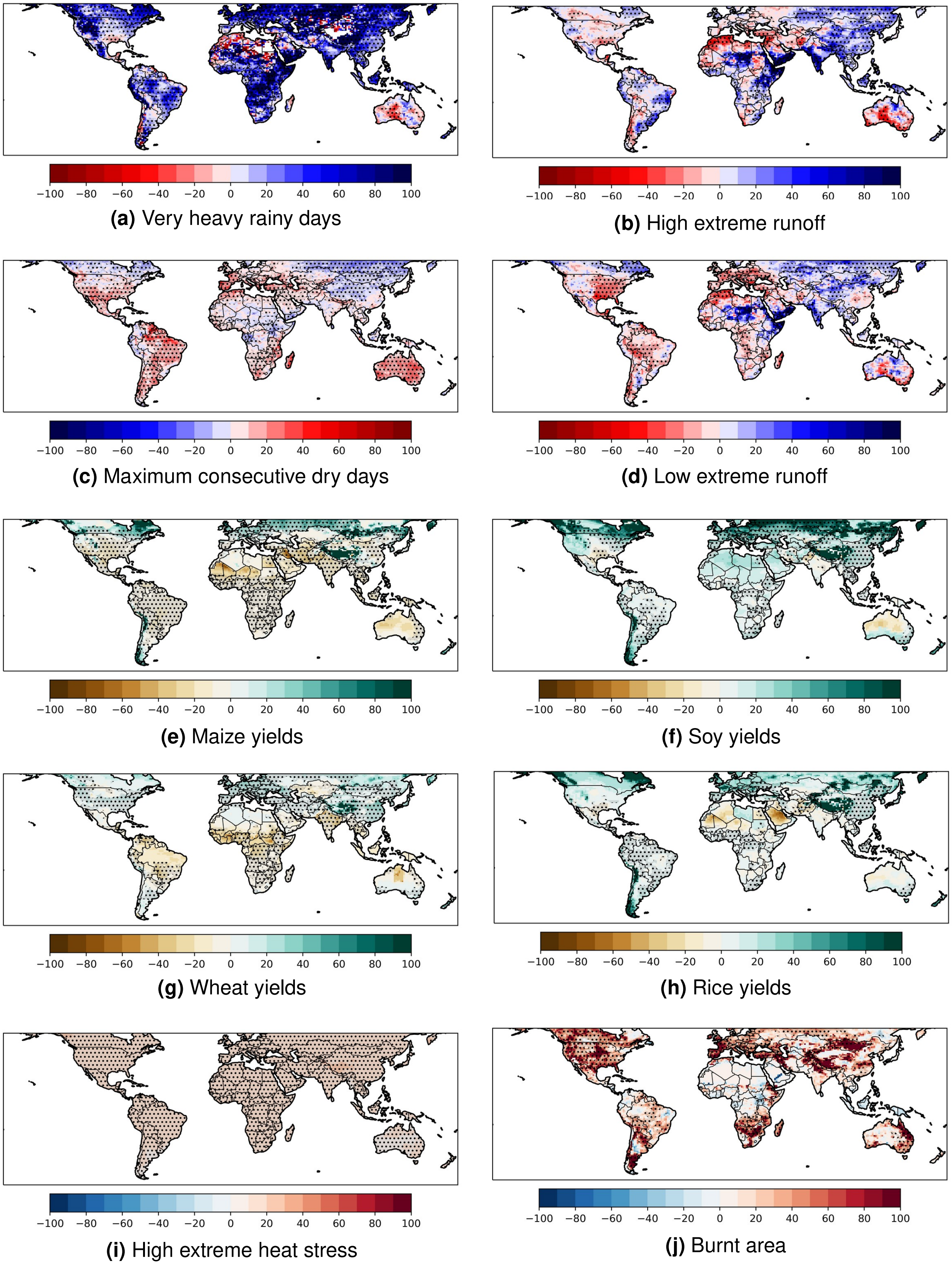

Fig. S1 . Spatial distributions of multi-model median projected changes of the 10 selected climate-related indicators under the RCP6.0 scenario. Dots indicate where at least 66% of per indicator GCMs × GIMs simulations agree. Each sign of the change corresponds to whether a stressor or a benefit depending on the indicator (Table 1 in main paper). Basemaps are based on OpenStreetMap (Open Database Licence) and Natural Earth (Public Domain).
