# Supplementary material for: Multiple climate-related stressors in the tropics and beneficial changes in northern latitudes will mostly have emerged before 2050
Source: PLoS One. 2025 Jun 17;20(6):e0293551. doi: 10.1371/journal.pone.0293551 (PMC12173232; doi:10.1371/journal.pone.0293551)
Supplement: S2 Fig — (PDF) [file pone.0293551.s004.pdf]

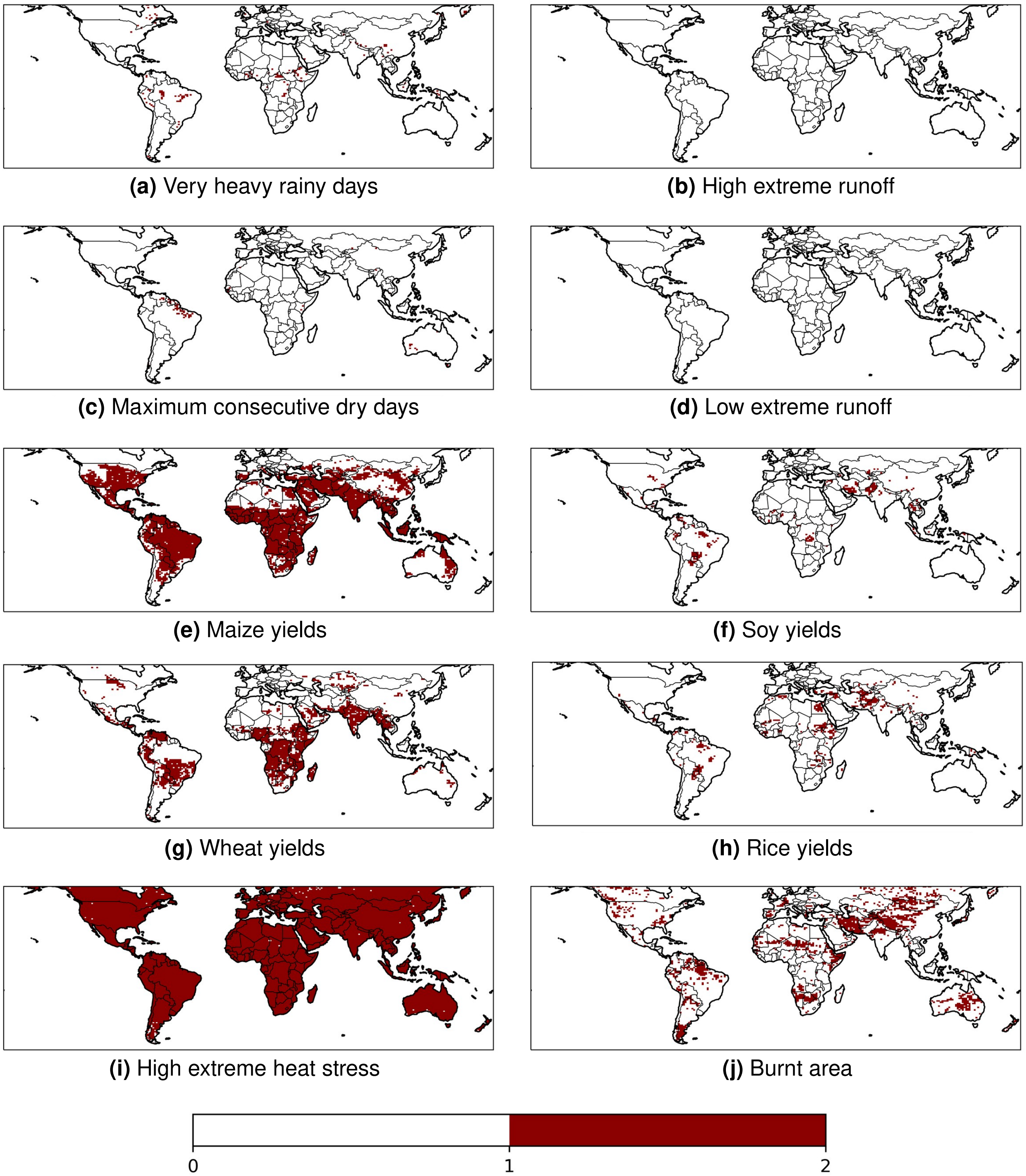

Fig. S2 . Spatial distributions of multi-model median projected <u>adverse</u> emergence for the 10 indicators under the RCP6.0 scenario. Values of 1 mean there are both (1) a future projected risk (each indicator change sign corresponds to a risk or a benefit depending on the indicator; main paper Table 1), and (2) a TOE detected for this sign change. Values of 0 mean there is no projected stressor and/or no detected TOE for this change sign. Basemaps are based on OpenStreetMap (Open Database Licence) and Natural Earth (Public Domain).
